# Intraoperative fluorescence perfusion assessment should be corrected by a measured subject-specific arterial input function

**DOI:** 10.1117/1.JBO.25.6.066002

**Published:** 2020-06-09

**Authors:** Jonathan T. Elliott, Rocco R. Addante, Gerard P. Slobogean, Shudong Jiang, Eric R. Henderson, Brian W. Pogue, Ida Leah Gitajn

**Affiliations:** a Dartmouth-Hitchcock Medical Center, Department of Surgery, Lebanon, New Hampshire, United States; b Thayer School of Engineering, Dartmouth College, Hanover, New Hampshire, United States; c University of Maryland School of Medicine, R Adams Cowley Shock Trauma Center, Department of Orthopaedics, Baltimore, Maryland, United States; d Dartmouth-Hitchcock Medical Center, Department of Orthopaedics, Lebanon, New Hampshire, United States

**Keywords:** indocyanine green, tracer kinetics, fluorescence, perfusion, angiography, arterial input function

## Abstract

**Significance:** The effects of varying the indocyanine green injection dose, injection rate, physiologic dispersion of dye, and intravenous tubing volume propagate into the shape and magnitude of the arterial input function (AIF) during intraoperative fluorescence perfusion assessment, thereby altering the observed kinetics of the fluorescence images *in vivo*.

**Aim:** Numerical simulations are used to demonstrate the effect of AIF on metrics derived from tissue concentration curves such as peak fluorescence, time-to-peak (TTP), and egress slope.

**Approach:** Forward models of tissue concentration were produced by convolving simulated AIFs with the adiabatic approximation to the tissue homogeneity model using input parameters representing six different tissue examples (normal brain, glioma, normal skin, ischemic skin, normal bone, and osteonecrosis).

**Results:** The results show that AIF perturbations result in variations in estimates of total intensity of up to 80% and TTP error of up to 200%, with the errors more dominant in brain, less in skin, and less in bone. Interestingly, error in ingress slope was as high as 60% across all tissue types. These are key observable parameters used in fluorescence imaging either implicitly by viewing the image or explicitly through intensity fitting algorithms. Correcting by deconvolving the image with a measured subject-specific AIF provides an intuitive means of visualizing the data while also removing the source of variance and allowing intra- and intersubject comparisons.

**Conclusions**: These results suggest that intraoperative fluorescence perfusion assessment should be corrected by patient-specific AIFs measured by pulse dye densitometry.

## 1 Introduction

Inadequate tissue perfusion is associated with a wide array of complications including infection,^1^ necrosis,^2^ osteomyelitis,^3^ functional deficits,^4^ and peritonitis,^5^ and it is responsible for a significant portion of unanticipated reoperations during postoperative recovery. Several fluorescence imaging systems are now currently deployed to assess perfusion intraoperatively by injection of indocyanine (ICG). These include, at the time of publication and to our knowledge: Stryker (formerly Novadaq) SPY PHI and SPY Elite, Zeiss Pentero FLOW800, Medtronic EleVision, Quest Spectrum, Fluoptics Fluobeam 800, Hamamatsu PDT Eye, Olympus Visera Elite II, and the OnLume Asimov-MKS Imaging System. These systems are FDA approved and indicated for fluorescence imaging of blood flow and tissue perfusion before, during, and after vascular, gastrointestinal, organ transplant, plastic, and reconstructive surgeries, as well as microsurgeries, allowing the surgeon to “visually assess circulation and related tissue perfusion.”^6^ This implies at least the possibility of, for example, comparing perfusion before and after modifying an anastomosis or addressing an arteriovenous malformation. However, to fully leverage the capabilities of this exciting new technology—to define parameter thresholds and make within-subject and between-subject comparisons of temporospatial data—a major source of variation must be addressed, mainly, the arterial input function (AIF).

The AIF is so-named because (a) it represents the time-dependent concentration of indicator dye in the arterial system that delivers blood to the tissue region-of-interest (ROI), and (b) it is an input function in the classic sense of linear time-invariant (LTI) systems. The subsequent section will describe, mathematically, the rationale for describing an ROI in this manner; for now, the reader is reminded that LTIs are so-called because they exhibit the same linear response (output) to an input irrespective of any previous inputs and their outputs for a linear combination of inputs are the same as a linear combination of individual responses to those inputs.^7^ The characteristic function of an LTI is called the transfer function, and given any input to the system, the output can be determined by convolving the input with this transfer function. Finally, if the LTI is a “black box” for which no explicit description is available, the transfer function can be determined by providing a delta function input—in this particular case, the system output is equal to the transfer function.

Most tracer kinetic techniques involve the introduction of a bolus of the tracer into the subject. Although a bolus injection may approximate a delta input for temporal behavior on the order of minutes, in “first-pass” high-temporal-resolution applications, it does not do so sufficiently. The injection rate, dispersion by the venous and pulmonary circuit, and recirculation time (cardiac output or venous return) all modify the temporal shape of the input.^8^^–^^11^ For this reason, established clinical methods such as CT perfusion and MR perfusion for stroke and tumor characterization^12^^–^^14^ require AIF characterization, typically by selecting pixels in an imaging plane that correspond to a large artery such as the carotid artery.^15^ Methods that recover compartment model parameters such as dynamic contrast-enhanced (DCE)-MRI of breast tumor may employ population AIFs measured using standard autoinjectors since $K_{\mathrm{trans}}$ and $k_{\mathrm{ep}}$ acquired over 3 to 5 min are less susceptible to intrasubject variation and the use of an average AIF only degrades the accuracy of recovered parameters slightly, compared with individually measure AIFs, if the AIF is matched to the patient age.^16^ However, this variability increases significantly when parameters such as blood flow (F), blood volume (BV), extraction fraction (EF), or permeability surface-area product are considered.^13^

Intraoperative fluorescence has evolved from other intraoperative imaging modalities such as angiography and fluoroscopy to assess patency of vessels,^17^^–^^20^ and therefore, it is designed to be employed in a qualitative real-time manner. In addition to patency of organ transplants, bypass grafts, free flap transfers, and vascular surgery procedures, assessment of lower extremity perfusion in peripheral artery disease and trauma have emerged as clinical applications of intraoperative fluorescence, and groups have employed qualitative^21^^,^^22^ and semiquantitative^23^^,^^24^ methods showing improvement in outcomes for several procedures. A retrospective review of patients who underwent breast reconstruction using ICG angiography, for example, reported a lower incidence and severity of skin necrosis and fewer reoperations for perfusion-related complications than patients who did not have ICG angiography.^25^ A prospective, multicenter clinical trial that evaluated the efficacy of intraoperative fluorescence angiography in left-sided colectomy and anterior resection (the PILLAR-II study) showed a marked decrease in the occurrence of anastomotic leaks. In 8% of the patients studied, real-time fluorescence visualization was used to modify the resection margin because the original margin did not adequately exclude all nonperfused tissue—a risk factor for anastomotic leak; none of these patients went on to have an anastomotic leak.^26^ More recently, a meta-analysis of 1177 patients, including those in the PILLAR-II study and three additional large studies, reported a lower rate of anastomotic leakage in patients who received ICG angiography (OR=0.27).^27^ When ICG angiography was used to assess free flap re-exploration (reoperation to salvage the flap after failure), its diagnostic ability to detect microvascular thrombosis was very high.^28^

None of the studies described above rely on quantification of fluorescence for improving clinical outcomes, rather, they involve visual inspection of tissue vascularity and enhancement associated with perfusion, inferred from real-time fluorescence video. This historical development path has de-emphasized quantification in favor of quick, easy to use, qualitative assessment. Nevertheless, a few groups have focused on enhancing the information available to the surgeon by analyzing the fluorescence image series acquired during ICG injection and washout and displaying maps of simple metrics. Matsui et al. presented a contrast-to-background ratio (CBR) time-curve approach: ROIs were divided by background and analysis was performed on the resulting CBR-curve.^29^ They report that metrics from this simple semiquantitative approach performed well diagnostically to identify viable and nonviable small bowel in rats. Diana et al.^30^^–^^33^ developed a fluorescence-based enhanced reality approach that uses time-to-peak (TTP) analysis to produce a perfusion-like map that is overlaid onto the surgeon’s view of the field and evaluated the approach in a variety of pig models. Similar TTP methods have also been evaluated in human patients.^34^ These approaches have advanced the quantitative nature of ICG fluorescence imaging and highlight the potential of quantitative curve analysis to provide additional information to the surgeon. However, none fully account for the intersubject variability of the AIF by directly measuring it, potentially diluting the diagnostic performance of the recovered metrics. In short, the full potential of this instrumentation to provide quantitative assessment of perfusion has not yet been realized.

The ability to obtain quantitative, kinetic parametric maps of the type measured by MR or CT perfusion, but intraoperatively and repeatedly before, during, and after intervention, could provide the surgeon with critical information to guide treatment. To this end and for the particular orthopaedic applications that we engage, our team developed a bone kinetic model that incorporates a measured AIF and demonstrated its use in a porcine model of tibia open fracture.^35^ This paper seeks to demonstrate the critical importance of AIF quantification in further refining and applying intraoperative fluorescence techniques to guide intervention and management of patients, even when simple curve analysis is used.

## 2 Materials and Methods

### 2.1 Indicator-Dilution Theory

If a tracer moves from the feeding arteriole to the draining veniole, through an organ or tissue bed of interest, then the flux of the tracer through that tissue of interest [dQ(t)/dt] is given by Fick’s principle:

$$\frac{dQ(t)}{dt}=FC_{a}(t)-FC_{v}(t),$$ (1)

where F is the blood flow, $C_{a}(t)$ is the time-dependent arterial concentration of dye, and $C_{v}(t)$ is the time-dependent venous concentration of dye. This principle forms the basis of indicator dilution theory. In the case of nondiffusible tracers such as indocyanine green, gadolinium contrast agents, or iohexol, the tissue is not a well-mixed compartment but rather is composed of microvessel units supplied by a single input and draining to a single output. The path that each individual tracer molecule takes is practically nondeterministic (“a black box”) but can be represented statistically by a distribution of transit times h(t) accounting for all possible paths through the capillary mesh. The venous output $C_{v}(t)$ is therefore mathematically the result of the arterial input $C_{a}(t)$ modified by h(t), or in other words, it is represented by the LTI system:

$$C_{v}(t)=\overset{t}{\underset{0}{\int }}C_{a}(u)h(t-u)du,$$ (2)

where u is the “bound variable” in the convolution between $C_{a}(t)$ and h(t). It is important to note that this convolution definition of indicator dilution behavior is not dependent on any modeling assumptions, aside from the presumption of anterograde single-input single-output flow. Since venous concentration is difficult to measure at the egress of the tissue of interest, we substitute Eq. (2), the definition of $C_{v}(t)$, into Eq. (1) and (using convolution notation, *) the Fick principle becomes:

$$\frac{dQ(t)}{dt}=FC_{a}(t)-FC_{a}(t)*h(t).$$ (3)

Closely related to the function h(t) is a more convenient function R(t) called the “impulse residue function,” which is also a fundamental description of the underlying dynamics of the system. R(t) is defined as the fraction of dye remaining in the tissue at time t following the injection of an idealized bolus (i.e., a Dirac delta function) and is given by

$$R(t)=1-\overset{t}{\underset{0}{\int }}h(u)du.$$ (4)

Integrating Eq. (3) and substituting in R(t) yields the convolution definition of indicator dilution:

$$Q(t)=C_{a}(t)*FR(t).$$ (5)

Since R(t) is a fundamental description of the tissue kinetics, it is the target of any kinetic analysis. Inferences about FR(t) can be made from Q(t) if and only if $C_{a}(t)$ is constant and reproducible on the relevant time scale of interest. For diffusible tracers (Xenon CT, $H_{2}O$ PET, etc.) that provide instantaneously well-mixed compartments or for late-phase dynamics such as extravascular leakiness, enhanced permeability and retention, or other cancer-related parameters ($K_{\mathrm{trans}}$, $K_{\mathrm{ep}}$ in DCE-MRI, and DCE-CT), intrasubject variability in the AIF is not likely to be a large source of error. However, for so-called first-pass kinetics in which the peak fluorescence intensity ($I_{\max}$), TTP, and slopes of ingress and egress [the former being also referred to as the blood flow index (BFI)] are measured, the AIF is of critical importance and in our estimation, has been underappreciated in the literature, especially in the field of intraoperative fluorescence perfusion, also called laser-assisted ICG angiography.

### 2.2 Simulation of Arterial Input Function

A gamma-variate AIF based on the formula published by Madsen^36^ but modified to include one recirculation pass was simulated using the following equation:

$$C_{a}(t)=y_{\max}t_{p}^{\alpha }e^{\alpha (1-t_{p})}+\frac{1}{10}y_{\max}t_{p}\prime^{\alpha /2}e^{\alpha (1-{t_{p}}^{\prime })/2},$$ (6)

where $t_{p}=t/t_{\max}$, ${t_{p}}^{\prime }=(t-\mathrm{TR})/4\;t_{\max}\Theta (t-\mathrm{TR})$, and Θ(t−TR) is a Heaviside function with transition at TR. Independent variables of Eq. (6) include $Y_{\max}$, the maximum concentration of the AIF, $t_{\max}$, the arterial TTP, α, a dispersion term, and TR, the recirculation time lag.

### 2.3 Part A: Error Analysis

#### 2.3.1 Perturbations to the arterial input function

**Fig. 1:**
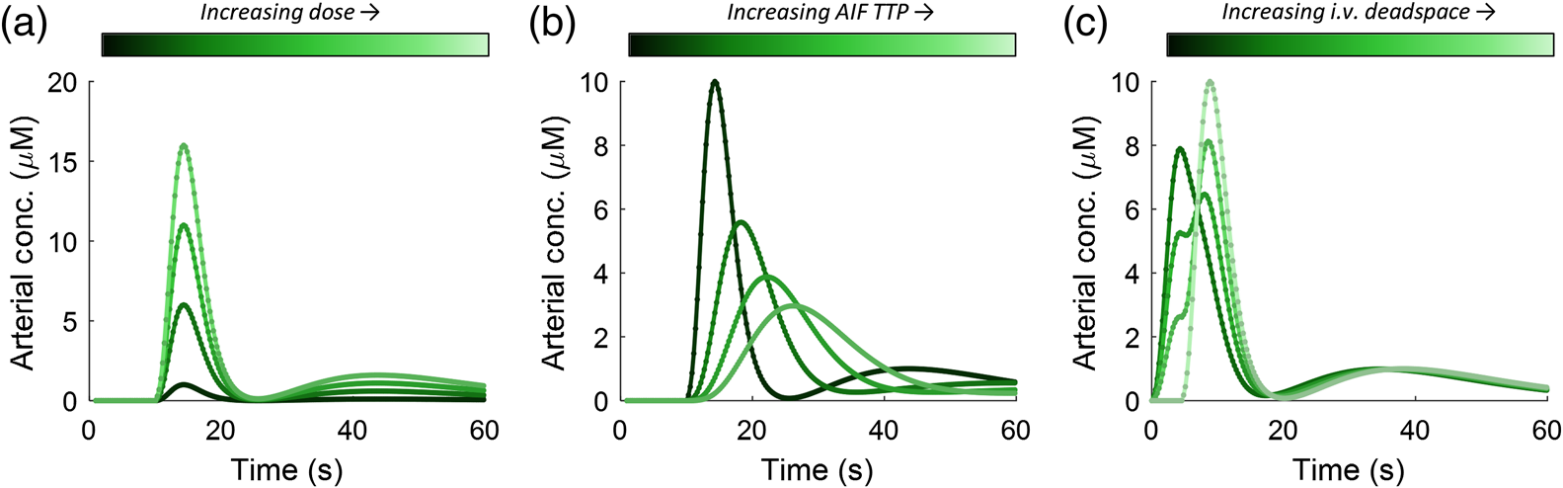
AIFs generated by modifying (a) dose, (b) speed of injection (TTP of the AIF), and (c) the “deadspace” in the IV tubing, which causes the bolus to enter the circulatory system in two phases.

All experiments were numerical in nature; no animal or human subjects are reported in this paper. We explored three main perturbation in the AIF based on our clinical experience as being most likely to occur and significant. The first perturbation was variation in AIF peak concentration, which can occur when the injected dose (i.e., number of ICG molecules) is varied or when the patient’s total BV is different. Patients are normally dosed according to weight, so variations in total BV as a function of weight (i.e., allometric variation) will introduce uncertainty.^37^ Previous studies have demonstrated that lean nonpregnant females have an average BV of 65  mL/kg, whereas obese nonpregnant females have an average BV of 45  mL/kg.^38^^,^^39^ There is also significant variation within these groups. Dose was varied from 0.1 to 2.0  mg/kg, being held at 1.0  mg/kg during other perturbations. The second perturbation that we explored was variation in the temporal width of the bolus expressed as the TTP of the AIF. This source of variation is introduced by the individual(s) performing the injection, as well as variations in dispersion that occurs when the bolus of dye is taken up by the heart from the venous system and pumped to the lungs and back, before being pumped to the tissue of interest. Arterial TTP was varied in the AIF model from 5 to 20 s, being held at 5 s during other perturbations. The peak of the AIF ($y_{\max}$) was normalized to the area under the curve, so the dose remained constant across all AIF TTPs. The third perturbation that we investigated was the amount of deadspace in the intravenous (IV) tubing used for the ICG injection, expressed as a fraction of the total injection volume. The marketing materials for indocyanine green used for intraoperative fluorescence state that the IV tubing volume must exceed the injection volume, so the ICG is first introduced into the IV tubing without entering the patient’s circulation and is then followed by a rapid saline flush of at 5 to 10 mL. If the ICG injection is larger than the IV tubing volume, the result will be a two-phase injection in which fraction a is injected, followed by a delay, and then fraction b=(1−a). This alteration of AIF shape can have profound effects on all parameters. The IV deadspace was varied from 1.0 to 0.0 as a fraction of injection volume, being held at 1.0 during other perturbations. Figure 1 shows the range of different AIFs produced by these perturbations.

#### 2.3.2 Simulation of tissue concentration curves

The numerical experiments included forward models to simulate the dynamic behavior of six different representative tissue types: brain, cortical bone, skin, glioma, osteonecrosis, and ischemic skin. These tissue types were selected based on the collective clinical and/or research expertise of the authors and the availability of experimentally derived typical kinetic parameters as listed in Table 1. The kinetic model used to generate the FR(t) functions is the adiabatic approximation to the tissue homogeneity (AATH) model first described by Lawrence and Lee.^40^^,^^41^ The AATH model decomposes the R(t) function into explicit vascular and parenchymal phases, which are temporally discretized and are defined as

$$R_{v}(t)=1-\Theta (t-t_{c}),$$ (7)

$$R_{p}(t)=Ee^{k_{ep}(t-t_{c})}\Theta (t-t_{c}),$$ (8)

$$Q(t)=C_{a}(t)*F[R_{v}(t)+R_{p}(t)],$$ (9)

where $t_{c}$ is the capillary transit time, E is the extraction fraction, $k_{\mathrm{ep}}$ is the rate transfer constant between the EES and IVS, and $\Theta (t-t_{c})$ is a Heaviside function with transition at $t_{c}$. Input parameters in Table 1 were selected from the literature^42^^,^^43^ and from our own clinical data (unpublished).

**Table 1:** Input parameters for the numerical simulation of six tissue types.

Tissue	Blood flow, F (mL/min/100 g)	Capillary transit time, $t_{c}$ (s)	Extraction fraction, E	Rate transfer constant, $k_{\mathrm{ep}}$ ($\min^{-1}$)
Normal tissue				
Brain	30	3.0	0.05	0.1
Cortical bone	5	12.0	0.5	0.01
Normal skin	5	5.0	0.1	0.1
Diseased tissue				
Glioma	10	12	0.15	0.01
Osteonecrosis	1	20.0	0.85	0.02
Ischemic skin	2	10.0	0.1	0.1

AIFs were generated using the above model, varying the independent variables of ICG dose, bolus injection width, and amount of IV “deadspace” as a fraction of the total injection volume. These were convolved with IRFs produced using the AATH model [(Eqs. (7)–(9)] and using the inputs given in Table 1 to produce corresponding tissue concentration curves. Gaussian white noise of 5% was added to each simulated curve and to the simulated AIFs.

#### 2.3.3 Recovery of perfusion-related parameters

**Fig. 2:**
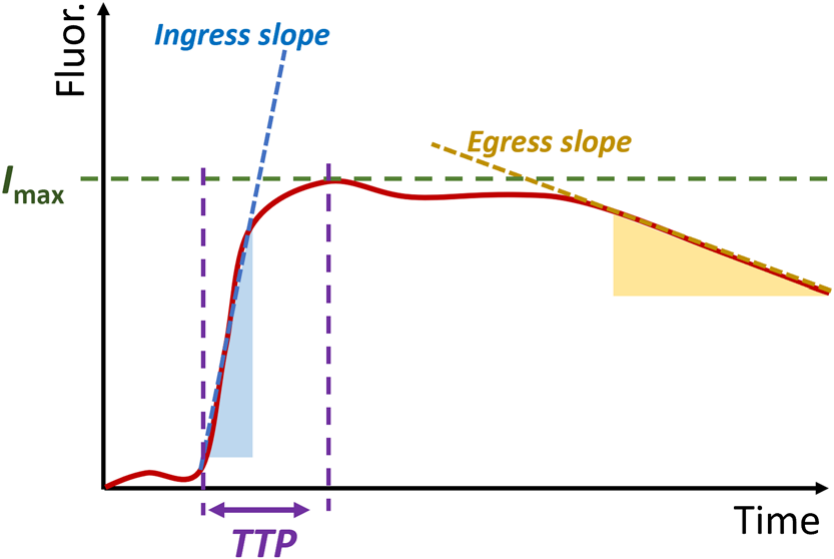
The simple curve analysis produces typically one or more of the following parameters: maximum fluorescence intensity ($I_{\max}$), TTP, ingress slope (also called BFI), and egress slope.

Perfusion-related parameters are derived from the simulated tissue concentration curves. In an image series, these parameters are obtained from the tissue time–concentration curve for each pixel or for an ROI averaged from multiple pixels (Fig. 2). Absolute and relative perfusion values are obtained at a specific time (1-min postinjection and 2-min postinjection) or a time corresponding to the peak of the whole image series (average tissue concentration curve). Arrival time and TTP are time-dependent parameters that are frequently obtained and visualized during microvascular procedures. Finally, ingress (BFI) and egress slopes are time and intensity-dependent, and were calculated by fitting a line to the portion of the curve between 10% and 90% of the maximum for ingress, and the portion of the curve below 90% of the maximum after peak, shown by the shaded regions in Fig. 2.

### 2.4 Part B: Correction by Mapping DCE-FI Data to Standardized AIF Space

To correct for AIF-dependent effects while maintaining the familiar and expected DCE-FI dynamics to which end-users have become accustomed, we propose mapping all DCE-FI data to a standardized AIF. The corrected dye concentration Q for a pixel r at time t is given by

$$Q(r,t)=C_{a}(r,t)*FR(r,t),$$ (10)

$$FR(r,t)=\mathrm{deconv}\{Q(r,t),C_{a}(t)\},$$ (11)

$$Q^{\prime }(r,t)={C_{a}}^{\prime }(r,t)*\mathrm{deconv}\{Q(r,t),C_{a}(t)\},$$ (12)

where ${C_{a}}^{\prime }(t)$ is a standard AIF (can be a population-average AIF or modeled using the AIF simulation method above), R(t) is the impulse response function, and Q(t) is the measured time–concentration curve. Deconvolution was performed using the truncated SVD approach (which imposes no physiological constraints) by taking the eigendecomposition of the Toeplitz matrix of $C_{a}(t)$ and computing the pseudoinverse of that matrix truncated to 11 singular values with the vector form of Q(t).^44^^,^^45^

## 3 Results and Discussion

### 3.1 Part A: Error Analysis of AIF Perturbations

**Fig. 3:**
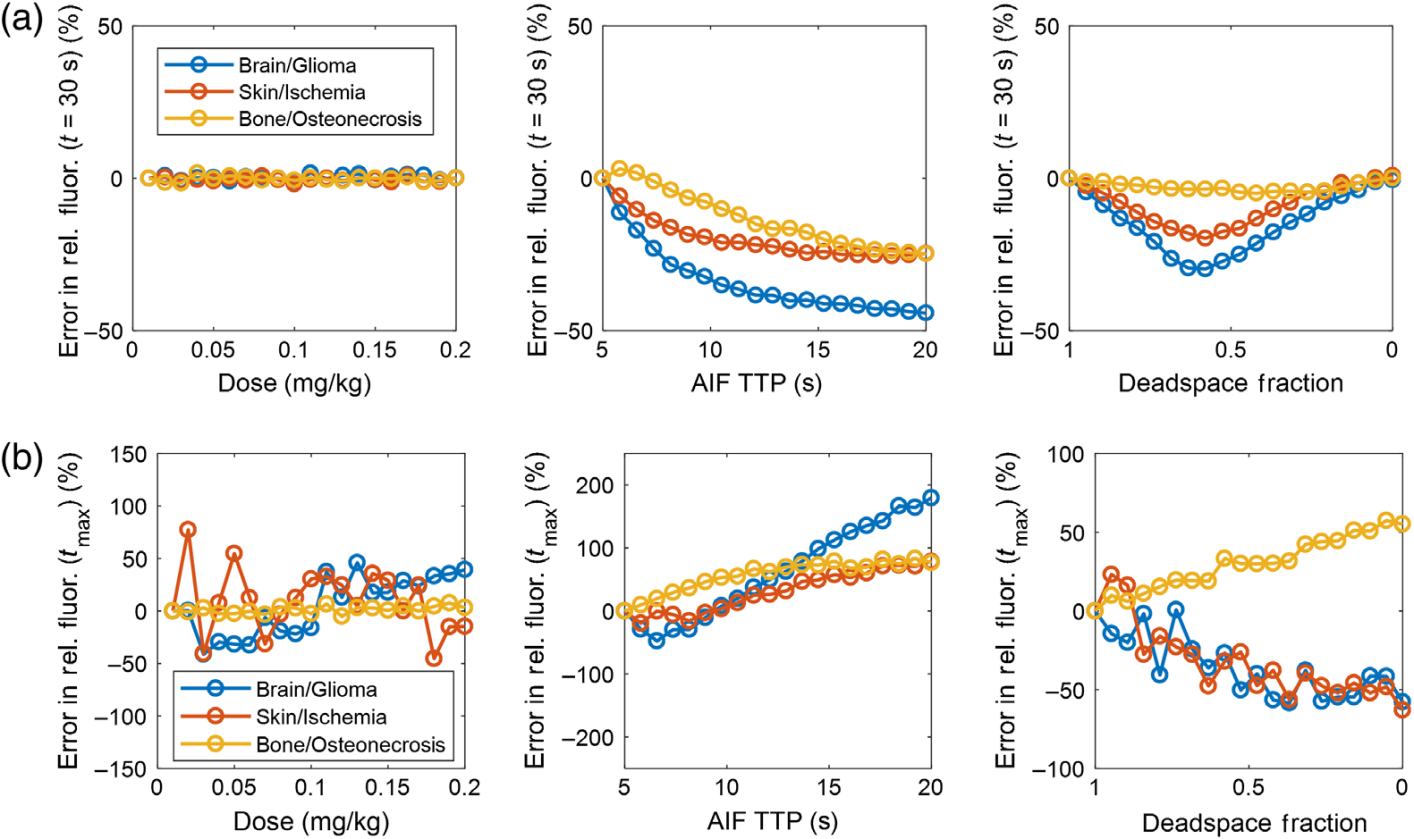
(a) Error in the relative fluorescence at an arbitrary timepoint of t=30  s. (b) Error in the relative fluorescence at the time of max fluorescence.

The first set of simulations demonstrate the effect of AIF perturbations on the relative max fluorescence parameter that is common in free flap evaluation, as well as a relative fluorescence metric defined at an arbitrary timepoint of 30 s after injection. In each case, the normal tissue (brain, skin, and bone) is used as the normalization ROI, and relative fluorescence is given for the diseased regions relative to normal. These simulations are summarized in Fig. 3. Although dose had no effect on this relative metric, AIF TTP did impact the relative metrics, as did the shape of the bolus as affected by IV deadspace. In these cases, the error can be attributed to the differential effect of AIF broadening on the normal and diseased tissue types.

**Fig. 4:**
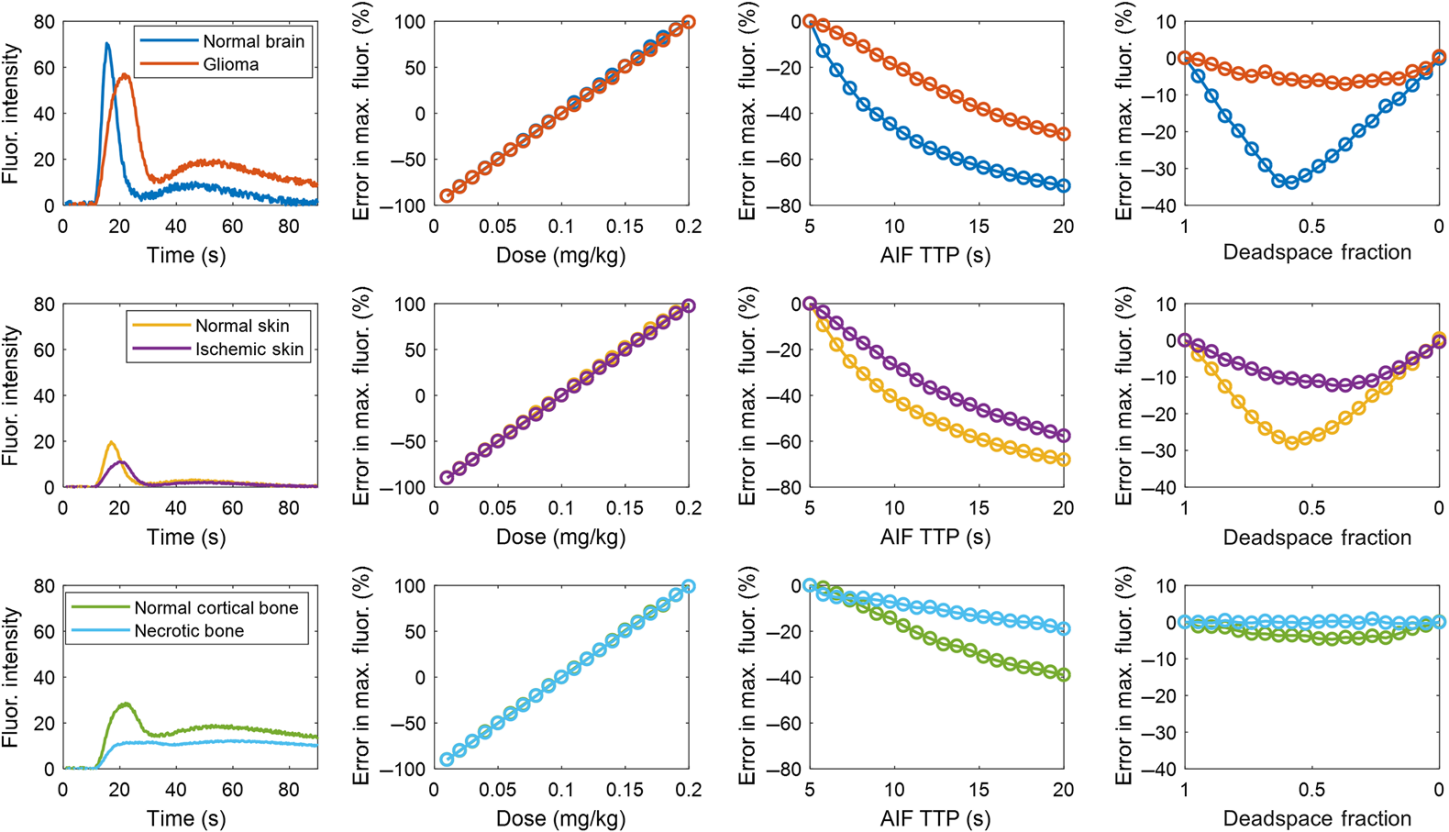
Error analysis of absolute fluorescence intensity in six different tissue types for a range of AIF perturbations in dose, AIF TTP, and IV deadspace fraction. First column shows the tissue concentration curves resulting from the standard input values. The analysis was carried out for three different tissue types, brain, skin, and bone (in each row, respectively), along with a possible disease state.

Figure 4 summarizes the effect of the three AIF perturbations on the maximum ICG fluorescence (i.e., the absolute value or sometimes called quantitative measurement). As expected, a dose-dependent error is observed for all tissue types. Slower tissue types were more dramatically affected by a variation in AIF TTP since these effects are exaggerated by their convolution with broader R(t) functions associated with these tissues of lower blood flow. However, conversely, maximum fluorescence was most dramatically impacted by the deadspace fraction in faster tissues, but less so in the slower tissues. This can be explained by the blurring effect of the convolution with slow tissue that mitigates the associated bimodal shape of the AIF.

**Fig. 5:**
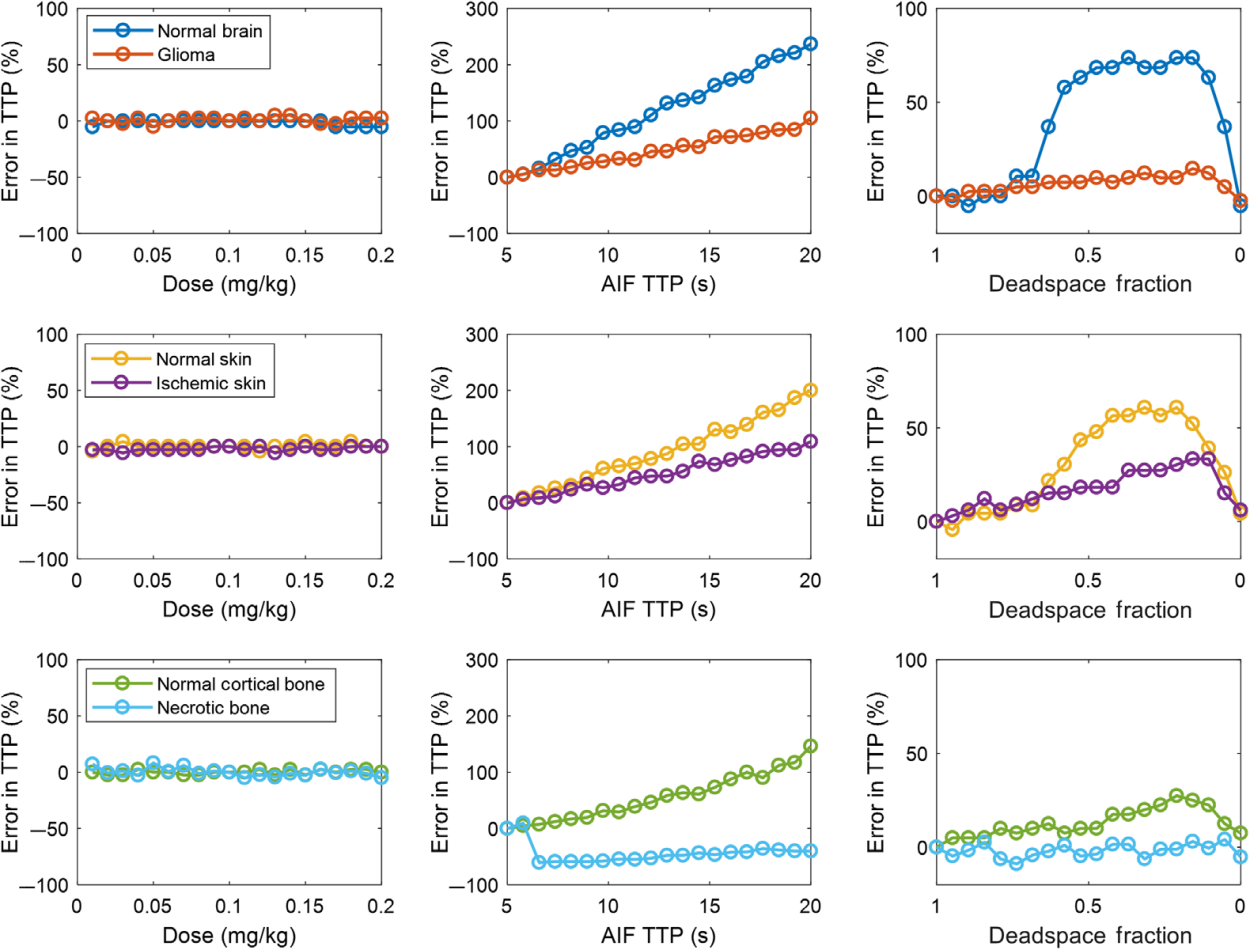
Error analysis of TTP in six different tissue types for a range of AIF perturbations in dose, AIF TTP, and IV deadspace fraction (in each column, respectively), for three different tissue types, brain, skin, and bone (in each row, respectively), along with a possible disease state.

The effect of the three AIF perturbations on the TTP metric is summarized in Fig. 5. For this metric, since it is entirely time-dependent, it is insensitive to dose. However, TTP was influenced by both AIF TTP and IV deadspace fraction. In all skin types except necrotic bone, TTP error increased linearly as a function of AIF TTP. The rate of error increase is a function of how broad the R(t) is for the specific tissue type. For the necrotic bone, a nonmonotonic relationship is observed. This is due to the fact that the inputs for this tissue type include a significant EF and rate transfer constant, as well as a long capillary transit time, and it is therefore the tissue type with the most bulk movement of dye from the intravascular space to the extravascular extracellular space.

**Fig. 6:**
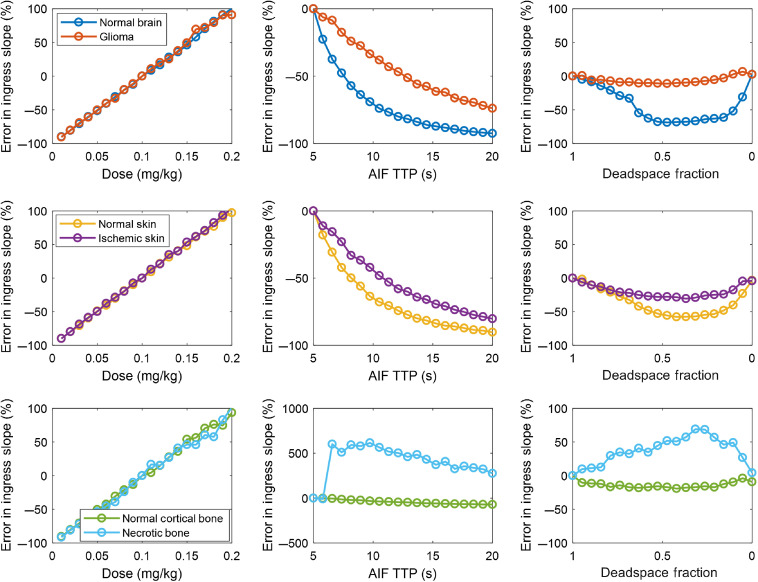
Error analysis of ingress slope (also called BFI) in six different tissue types for a range of AIF perturbations in dose, AIF TTP, and IV deadspace fraction.

Figure 6 depicts the error in ingress slope—also called BFI—caused by the perturbations in AIF. Dose-dependent effects similar to maximum fluorescence are observed since the numerator of the slope is related to the maximum intensity. Temporal changes in the AIF also affect the recovered parameter, this time having the opposite effect by increasing the denominator. The deadspace fraction had profound effects as well.

Taken together, Figs. 3–5 demonstrate that substantial variation exists in relative and absolute fluorescence time to peak and ingress slope, with the more complex metrics being affected to a greater degree. Because even relative fluorescence is prone to errors caused by AIF variability, any comparisons either within a subject by serial ICG injections or between subjects in the form of a clinical threshold should be computed only after correcting for the effect of AIF variation.

### 3.2 Part B: Deconvolution/Reconvolution Correction Method

**Fig. 7:**
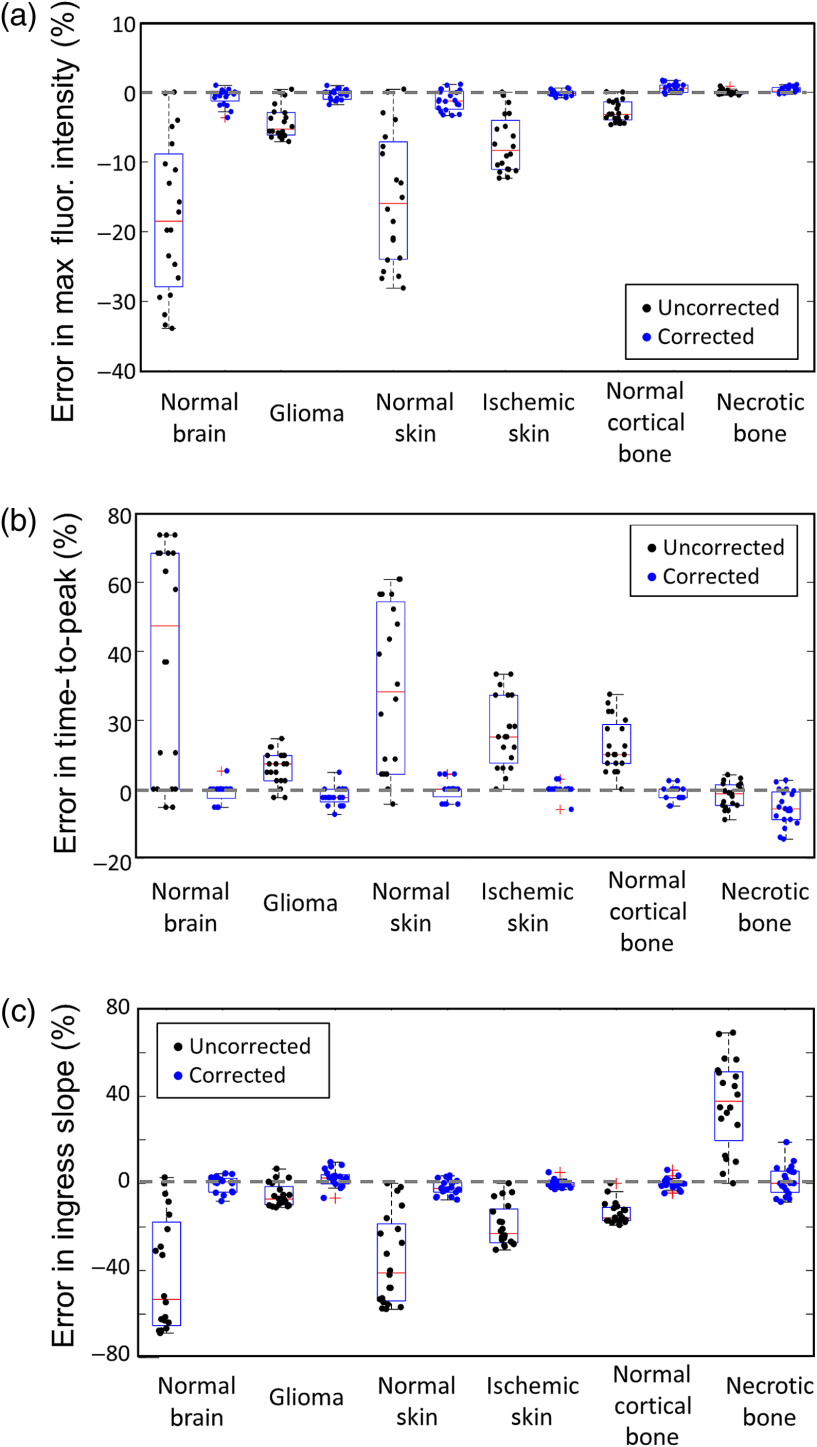
Error in (a) maximum fluorescence intensity, (b) TTP, and (c) ingress slope when the IV “deadspace” fraction is varied. Boxplots (min, first quartile, median, third quartile, max) represent the errors calculated from the values obtained across the entire range of deadpace fractions from 0 to 1.0, for each of the six tissue types. The values obtained after fitting the corrected tissue-concentration curves (blue dots) are compared with those obtained from the uncorrected curves (black dots).

Each parameter that was evaluated in the error analysis above was also corrected using the deconvoltion/reconvolution approach described in Eqs. (10)–(12). This approach deconvolves the FR(t) function from the measured Q(t) and AIF and then reconvolves it with a standard AIF to produce a corrected Q(t). The same simple curve analysis metrics are thus calculated from this corrected Q(t), for which the variations in AIF have been removed. Figure 7 summarizes the impact of this correction scheme for perturbations in IV fraction. Each dot represents the error from a single forward simulation used in the previous section. Black dots are uncorrected parameters and blue dots are corrected parameters. In every tissue type and across all three parameters, AIF correction substantially reduced the error. Greater than 95% of the corrected values had an error of less than 5%.

Several studies have recently been published demonstrating the utility of intraoperative fluorescence in assessing hemodynamics of tissue to guide treatment. Absolute maps in FUs were used to assess pre- and postdebridement perfusion and assess tissue viability.^22^ ICG angiography was used in a case study to provide rapid qualitative assessment of the efficacy of intra-arterial nitroglycerin, providing evidence of revascularization when pre- and post-treatment images were compared.^21^ Quantitative metrics—such as time to 50% of the uptake curve, $T_{1/2}$—obtained from measurements in peripheral arterial disease correlated with traditional assessment methods,^23^ but in critical limb ischemia, the same metrics showed large variations in the arrival time of dye,^24^ suggesting that the predictive power of these methods may be influenced by arterial delivery variability. Intraoperative imaging of breast implants after nipple-sparing mastectomy revealed improved ingress and egress rates in infra-areolar incision compared with supra-areolar incision—two different incision methods in this procedure.^46^

It is important to note that this paper, and in particular the deconvoluton/reconvolution correction method, is predicated on the idea that the AIF can be collected from patients in a straightforward way. In fact, the single biggest obstacle to refining kinetic analysis of intraoperative fluorescence is the lack of available dye densitometers (PDDs)^47^—the term given to the modified pulse oximeters that are sensitive to ICG and can characterize the arterial system specific dye concentration. Although systems are available in some markets for commercial purchase, no such devices are marketed in the United States. We have used the approach described by the team of the University of Pennsylvania and Western University^48^ in which a pulse dye densitometer is assembled using an integrated analog front-end for pulse oximeter evaluation board (AFE4490SPO2EVM, Texas Instruments, Dallas, Texas) and the standard probes (typically employing 660 and 940 nm modulated LEDs) are replaced with an ICG-sensitive probe employing 805 and 940 nm LEDs (TL-301P, Nihon Kohden, Tokyo, Japan). Software employing denoising and bandpass filtering to extract the pulsatile components have been developed in house and are being used to obtain an AIF from the digitized signal stored by the AFE4490 board. The clinical investigations currently underway at Dartmouth-Hitchcock (NCT04245111 and NCT04250558) are using patient-specific AIFs in quantitative ICG fluorescence perfusion assessment. Since these are observational only, it is convenient to extract the AIF and correct the ICG fluorescence images offline. Future interventional studies examining the effect of quantitative ICG imaging on patient outcome will require real-time intraoperative deconvolution/reconvolution and, therefore, real-time AIF acquisition.

Therefore, integration of pulse dye densitometry into imaging devices is a logical next step to improving the efficacy of intraoperative fluorescence. We recommend that imaging device manufacturers consider developing the next generation of devices to include a panel-mount electrical connector socket (e.g., DE-9 or circular DIN) that accepts a modified pulse-oximeter-style finger probe plug in future devices (optimized for ICG absorption at 805 nm). An analog front-end chip like the TI AFE4490 could be directly interfaced with the internal computer of the imaging system through a serial peripheral interface protocol with minimal effort. During “ICG acquisition mode,” the pulsatile component of the signal could be extracted and converted to an AIF. Deconvolution/reconvolution correction could then be applied to the data before it is displayed in near real-time (using a GPU to parallelize the operation since each pixel is independently corrected) and overlaid onto the surgeon’s view.

## 4 Conclusion

The error analysis presented in this paper shows that variation in the height, width, and shape of the AIF of the degree normally found in clinical practice can have important effects on the accuracy of both simple, relative parameters and more complex quantification such as ingress slope or TTP. However, when the AIF is acquired during measurement, a simple deconvolution/reconvolution correction scheme can be used to map the tissue concentration curves into those that would have resulted from a standard AIF. This approach mitigates any perturbations in the AIF, and recovered parameters show substantially less variability. We hope that the increased efforts toward quantification in intraoperative fluorescence assessment of perfusion, including this paper, will motivate the integration of pulse dye densitometer technology into the next generation of commercial devices.
